# Harnessing Endogenous Cellular Mechanisms for Bone Repair

**DOI:** 10.3389/fbioe.2017.00052

**Published:** 2017-09-04

**Authors:** Claudia Lo Sicco, Roberta Tasso

**Affiliations:** ^1^Department of Experimental Medicine, University of Genoa, Genoa, Italy; ^2^Ospedale Policlinico San Martino, Istituto di Ricovero e Cura a Carattere Scientifico per l’Oncologia, Genoa, Italy

**Keywords:** inflammation, injury microenvironment, bone repair, endogenous progenitor cells, circulating progenitor cells, skeletal progenitor cells, regenerative medicine

## Abstract

Although autologous tissue transplantation represents a valid approach for bone repair, it has encountered crucial barriers in therapeutic translation, not least the invasive process necessary for stem cell isolation. In recent years, the scientific community has made significant strides for identifying new treatment options, and great emphasis has been placed on the tight interaction between skeletal and immune system in modulating the outcome of bone repair. Within the context of specific injury environmental cues, the cross talk among inflammatory cells and tissue resident and/or circulating progenitor cells is crucial to finely coordinate repair and remodeling processes. The appropriate modulation of the inflammatory response can now be considered a new trend in the field of regenerative medicine, as it raises the attracting possibility to enhance endogenous progenitor cell functions, finally leading to tissue repair. Therefore, new treatment options have been developed considering the wide spectrum of bone–inflammation interplay, considering in particular the cell intrinsic cues responsible for the modulation of the injured environment. In this review, we will provide a panoramic overview focusing on novel findings developed to uphold endogenous bone repair.

## Introduction

Bone regeneration represents a series of biological events orchestrated by a large number of mediators and cellular elements leading to cell recruitment, proliferation, and differentiation (Einhorn and Gerstenfeld, 2014). Most skeletal fractures heal in the first 8 weeks without major clinical concerns (Marsell and Einhorn, 2011). However, in the case of impaired bone healing, fractures can be associated with a range of complications (Kostenuik and Mirza, 2017). Conventionally, if no healing is detectable after 4 months, the fracture can be considered a delayed union. If the failure of the fracture to consolidate persists for more than 6 months, it can be considered a non-union (Marsh, 1998). The dynamic process underlying bone healing involves the interactions of cells, cytokines and matrix and requires concerted events, consisting of early inflammatory response, hard callus formation, and bone union followed by remodeling (Giannoudis et al., 2007; Gómez-Barrena et al., 2015). Bone autograft is the harmless and most efficient grafting procedure. However, due to limitations related to quantity and harvesting, “it represents an additional surgical intervention, with frequent consequences of pain and complications” (Roberts and Rosenbaum, 2012; Gómez-Barrena et al., 2015). Having said that, the scientific community moved on to allograft, primarily hailing from tissue banks. Nevertheless, also this strategy could be impaired by virus-inactivation treatments and freezing procedures (Gómez-Barrena et al., 2015). Thus, it has been long searched for biocompatible materials, in combination or not with osteogenic factors, resembling the properties of the autografts (Giannoudis et al., 2005), but none of them has reached the same osteogenic potential. In view of these limitations, cell therapy can be considered an effective alternative to bone grafting (Rosset et al., 2014). Until now, different osteoprogenitors have been used in combination with suitable scaffolds. However, the application of these approaches led to a limited clinical success due to several reasons, such as the high commercialization costs, the regulatory issues, as well as the hitches of clinical translation (Bruder and Fox, 1999; Amini et al., 2012).

Among the most important biological interactions involved in the bone healing process, the cross talk between skeletal and immune system has received great attention, enough to establish an interdisciplinary field named osteoimmunology (Greenblatt and Shim, 2013). The appropriate modulation of the inflammatory response that occurs following tissue injury can be considered an important regulator of the bone repair cascade, leading to activation, mobilization, and recruitment of osteoprogenitors to the injured sites (Mountziaris and Mikos, 2008). In light of such considerations, the search is now on identifying the finest strategies to enhance and potentiate endogenous regenerative events for future therapy.

In this review, we will discuss the latest and most relevant findings on multiple features that impact fracture healing, with particular emphasis on the role of inflammation and progenitor cell recruitment.

## Inflammatory Responses Induced by Fracture Healing

Platelet activation and concurrent inflammatory reaction are the first tissue responses to damage. The sum of biological effects generated during these early phases will direct the entire healing process (Tasso et al., 2013). It is known that “a brief and highly regulated secretion of pro-inflammatory cytokines at the time of the acute injury is crucial for the healing process” (Marsell and Einhorn, 2011). Bone fracture leads to blood vessel disruption not only inside bone, but also in the adjacent soft tissues, and to a generalized damage of cells and tissues that, as a whole, induce a strong inflammatory reaction (Claes et al., 2012). The acutely inflamed surrounding tissues are characterized by vasodilatation and fast arrival of innate immune cells. Within the fracture gap, fibrinogen is converted in fibrin and the hematoma takes shape (Claes et al., 2012). The resulting environment is highly hypoxic and marked by low pH and strong infiltration of inflammatory cells and cytokines (Einhorn and Gerstenfeld, 2014). In this context, literature data indicate that interleukin-6 (IL-6) and tumor necrosis factor-alpha (TNF-α) play key roles in the regulation of osteoclast activity by stimulating hematopoietic progenitor cells to differentiate along an osteoclastic lineage or enhancing the resorptive capacity of existing osteoclasts (Sarahrudi et al., 2009; Yokota et al., 2014). The idea that a certain degree of inflammation is required is reinforced by literature data indicating that treatment with anti-inflammatory drugs such as cyclooxygenase 2 (COX-2) inhibitors impairs the fracture healing process (Claes et al., 2012). Indeed, COX-2 promotes both angiogenesis and differentiation of mesenchymal stem cells (MSCs) into osteoblasts during fracture healing (Boursinos et al., 2017). To back this up, in 2011 Liu and colleagues have demonstrated that the *in situ* administration of the non-steroidal anti-inflammatory drug aspirin significantly promoted MSC-mediated bone repair in a mouse model of calvarial defect (Liu et al., 2011b). Moreover, the inhibition of NF-kB, a transcription factor involved in inflammation, or pathways leading to its activation improved “MSC-mediated craniofacial bone regeneration and repair *in vivo*” contrasting β-catenin degradation (Chang et al., 2013). The involvement of the β-catenin pathway in bone repair was further demonstrated in a recent paper indicating that signaling associated with the danger molecule interleukin-1 receptor, type 1 (IL-1R1) impaired MSC activation and differentiation by inhibiting β-catenin pathway (Martino et al., 2016).

**Figure 1:**
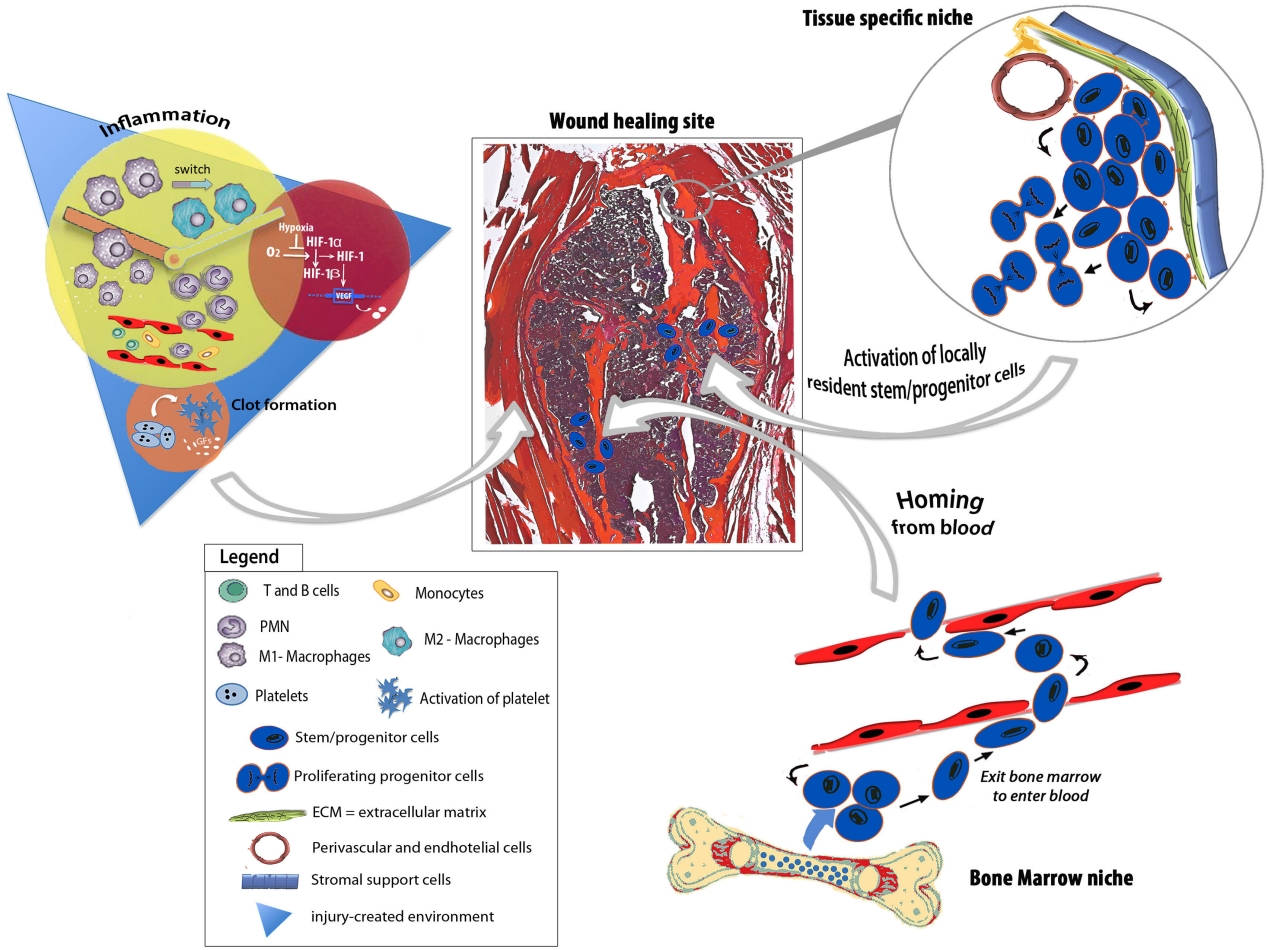
Schematic depicting endogenous regenerative responses underpinning bone tissue repair. The injury-generated environment is enriched of growth factors, chemokines, pro- and anti-inflammatory cytokines as well as hypoxic stimuli that evoke feedbacks to the niches, deploying programs for endogenous progenitor activation, mobilization, and recruitment to the healing site. The healing process involves either circulating progenitor cells or tissue-specific progenitors.

The reason why the initial cell interactions are essential to obtain a correct repair is that innate immune cells guide revascularization and reparative events at injury sites, promoting progenitor cell migration (Figure 1). In 2012, a study was conducted to evaluate how biomaterials designed to incorporate inflammatory signals affected the behavior of natural killer (NK) cells, one of the first population arriving at the injury site, and the NK/MSC interactions. “It was found that NK cells are capable of stimulating a three-fold increase in human bone marrow MSC invasion, suggesting the importance of designing novel biomaterials leading to rational modulation of the inflammatory response as an alternative to current bone regeneration strategies” (Almeida et al., 2012).

During the early inflammatory phase, polymorphonuclear leukocytes followed by blood monocytes/macrophages entrapped in the fibrin cloth release molecules that favor the chemoattraction of different cell types. Due to their plasticity, macrophages represent one of the most studied innate immune cell populations (Mantovani et al., 2013). Classically activated, or M1, macrophages, whose prototypical activating stimulus is interferon-gamma (IFN-γ) and TNF-α (Varga et al., 2016) exhibit potent antimicrobial properties, high capacity to present antigen, and high interleukin-12 (IL-12) and IL-23 production (Verreck et al., 2004). In response to IL-4 and IL-13 signaling pathways, macrophages undergo an alternative activation, or M2, program that takes part in polarized Th2 responses, dampening of inflammation, promotion of tissue remodeling (Wynn, 2004), and angiogenesis (Chambers et al., 2013). Recently, it has been suggested a more appropriate nomenclature for macrophages based on “a set of standards encompassing three principles—the source of macrophages, definition of the activators, and a consensus collection of markers to describe macrophage activation—with the goal of unifying experimental standards for diverse experimental scenarios” (Murray et al., 2014). Different studies indicate that the interaction with *bona fide* stem/progenitor cells is an important task of macrophages, and this is particularly true when these cells are in a M2 activation state (Lolmede et al., 2009; Tasso et al., 2013). It has been reported that “depletion of macrophages led to early skeletal growth retardation and progressive osteoporosis” (Vi et al., 2015). Resident- and circulating macrophages recruited to the injury site exert pivotal functions for intramembranous and endochondral ossification, respectively (Claes et al., 2012). *In vivo* depletion of resident macrophages, named osteomacs, indicated that these cells were required for deposition of matrix expressing type I collagen and bone mineralization (Alexander et al., 2011). Conversely, when the fracture healing process was examined in CCR2−/− mice that underwent a non-stabilized tibial fracture that heals through robust endochondral ossification, an impaired vascularization and decreased callus formation associated with a lower number of circulating macrophages were observed (Xing et al., 2010). The functional effect of macrophages on fracture healing has been recently confirmed using a preclinical model of osteotomy induced in rats subjected to splenectomy (Xiao et al., 2017). Moreover, studies conducted with human fracture tissues indicate that the presence of macrophages persists in the injured sites in association with areas of bone formation, even if their numbers are higher in early fracture samples (Andrew et al., 1994; Alexander et al., 2017). Overall, these data suggest that macrophage contribution to bone repair goes far beyond the early inflammatory events. Noteworthy, some recently published papers stressed the closed relationship between the adaptive immune system and the healing outcome. Mice totally lacking the adaptive immune system, as well as mice depleted of CD8+ T lymphocytes are characterized by an enhanced endogenous fracture regeneration (Toben et al., 2011; Reinke et al., 2013).

The milieu induced by the initial inflammatory response together with the angiogenic factors released as an effect of the hypoxic condition generated within the fracture gap (Mirhadi et al., 2013), guide revascularization of the injured site, a central event not only for the re-establishment of a normoxic environment, but also because the newly formed blood vessels allow the direct interaction with host cells and provide the access to host osteoprogenitor cells, enhancing matrix deposition.

## Endogenous Progenitor Cells in Bone Repair: Locally Resident Versus Circulating Progenitors

It has been described that signals associated with the environment generated by an injured bone stimulate the mobilization and proliferation of both resident and circulating progenitor cells needed for tissue repair (Hadjiargyrou and O’Keefe, 2014) (Figure 1). As early as 1965, a paper published on Science by Urist (1965) indicated that the process leading to new bone tissue formation was strictly related to the interactions with endogenous host cells and that differentiation of osteoprogenitors was elicited by local environment signals.

Just like different types of bone damages lead to different inflammatory cascades and, consequently, to different ossification processes (Colnot et al., 2012), also the involvement of progenitor cells is affected. When a fracture occurs in the absence of stabilization, a strong periosteal reaction takes place and the repair process brings about the formation of a large callus formed through an endochondral ossification process (Le et al., 2001; Chang and Knothe Tate, 2012). On the contrary, when fractures are firmly stabilized, the periosteal reaction is not efficient, and a minimal callus formation is driven by an intramembranous ossification process (van Gastel et al., 2014). In line with these concepts, holding over stabilization during the early phases of fracture healing does not affect either the volume or the mechanical properties of the callus, but it induces the formation of more cartilage, thus altering the involvement of endogenous progenitors and the modality of bone repair (Miclau et al., 2007). In this context, literature data indicate that different types of fractures cause changes in the expression of inflammatory genes, including matrix metalloproteinase 9 (*MMP9*), and in the subsequent activation and differentiation of resident periosteal progenitors (Wang et al., 2013). Periosteum-derived mesenchymal progenitors are vital for both endochondral and intramembranous cortical bone formation (Hutmacher and Sittinger, 2003). The contribution of periosteal progenitors to callus formation was examined using a mouse model of Rosa26 segmental bone graft transplantation (Zhang et al., 2008; Colnot et al., 2012). The study demonstrates that periosteal progenitors contribute to the initial phases of new bone formation, suggesting that this resident cell population act as an essential trigger for bone repair processes. Indeed, periosteal progenitors can act directly, differentiating to cartilage/bone tissue or indirectly, releasing osteoinductive factors that recruit and activate other host-osteoprogenitors (Zhang et al., 2005), although the relative contribution of these two mechanisms is still not clear. In 2009, it has been clearly demonstrated that periosteum and endosteum, the main sources of resident progenitors, differently participated to bone repair processes. To back this up, periosteal progenitors show a dose-dependent migratory effect under chemokine receptor ligands stimulation, such as CXCR4 and CXCR5 (Ferretti and Mattioli-Belmonte, 2014). Recently, using a combination of markers including AlphaV integrin, Chan et al. (2015) have defined a skeletal stem cell population present in the proximity of the growth plate of long bones and capable of differentiating bone, cartilage, and stroma *in vivo*. Meanwhile, another study highlighted the existence of Gremlin-1 osteochondroreticular (Grem1-OCR) stem cells concentrated within the metaphysis of long bones contributing to bone healing and possessing the ability to self-renew after serial transplantations (Worthley et al., 2015).

The severity of fracture healing is directly proportional to a reduced soft tissue envelope and “increasing severity of the surrounding muscle is associated with the development of non-unions” (Friedrich et al., 2011; Papakostidis et al., 2011; Shah et al., 2013). In light of these observations, muscle has been considered a “potential source of cells and signals for bone healing” (Shah et al., 2013). A recent study conducted in mice using an elegant cell lineage tracing approach has demonstrated that muscle precursor cells participate in callus formation only in the case of open fractures with periosteal stripping and muscle injury (Liu et al., 2011a). Related evidence further indicate that muscle-derived cells present within the fracture callus shift their gene expression from the muscle marker Paired Box Gene 3 (Pax3) to the chondrogenic markers SRY-Box 9 (Sox9) and Homeobox protein Nkx3 (Nkx3) (Cairns et al., 2012).

In addition to locally resident osteoprogenitors, other skeletal progenitors have been proposed to participate in bone repair processes (Pignolo and Kassem, 2011), including circulating bone marrow-derived progenitors (Table 1). However, to date, a precise comprehension on the signals released from the injured tissues responsible for the mobilization of bone marrow-derived progenitor cells and the accurate molecular mechanisms governing their fate, homing, and engraftment are very limited. Moreover, “it’s likely that individual circulating progenitors detected by different experimental strategies are overlapping but indicated with different names, such as circulating osteoprogenitors (COPs), alkaline phosphatase-positive (ALP+) circulating progenitors, circulating CD34-positive (CD34+) precursors, contributing to increase the confusion regarding their exact identification” (Lo Sicco et al., 2015).

**Table 1:** Resident and circulating progenitors involved in adult bone repair.

	Name	Markers	>Localization	Activation stimuli	Reference
**Resident progenitors**	Periosteal stem/progenitors	Sca-I+ CD105+ SSEA-4+ CD29+ CD140+	Periosteum	Hedgehog (Hh) signaling pathway	Wang et al. (2013)
	Bone, cartilage, stromal progenitor	CD45− Ter119− Tie2− AlphaV+ CD105+ CD200+	Growth plate	Hh. BMP. FGF, and Notch signaling pathways	Chang et al., (2013), Chan et al. (2015)
	Osteochondroreticular stem cells	CD45− Ter119− CD31− Grem1+	Growth plate and trabecular bone	BMP signaling pathway	Worthley et al. (2015)
**Circulating progenitors**	Connective tissue progenitors	ALP+	Peripheral blood	Injury-associated signals	Kumagai et al. (2008)
	Circulating osteogenic precursors	CXCR4+ CD44+ CD45−	Peripheral blood	BMP-2 signaling pathway	Otsuru et al. (2007)
	Myeloid CD34+	CD34+ OC+	Peripheral blood	SDF-1 signaling pathway	Matsumoto et al. (2008)
	Human osteoblast lineages cells	OC+ BAP+	Peripheral blood	BMP signaling pathway	Eghbali-Fatourechi et al. (2005)
	Circulating healing cells	Lineage− CD45−	Peripheral blood	Injury-associated signals	Lo Sicco et al. (2015)

So far, a population of adherent fibroblast-like cells with osteogenic potential has been isolated from the blood of different species, indicating that “cells with multiple differentiation potential analogous to that of post-natal marrow stromal cells can negotiate the circulation” (Kuznetsov et al., 2001). A first proof-of-principle set of experiments elucidating the differentiation potential of circulating CD34-positive (CD34+) cells into not only endothelial cells but also osteoblasts goes back to 2008 (Matsumoto et al., 2008). These cells were described to create a milieu favorable to a functional recovery from fracture. In the same year, taking advantage of parabiosis experiments, it was demonstrated that the injury-associated signals triggered by bone fracture induced a stimulus for recruitment of circulating alkaline phosphatase-positive (ALP+) cells, although the exact origin of recruited cells remains uncertain (Kumagai et al., 2008). More recently, a similar parabiotic approach was adopted to reveal that the exposure to a youthful circulation affected bone repair through the modulation of β-catenin (Baht et al., 2015), raising the possibility that agents that modulate this pathway could improve the extent and quality of fracture repair in the aging population. In 2015, the existence of a rare and undifferentiated cell population—circulating healing (CH) cells—involved in bone tissue healing and present in the peripheral blood of immunocompetent mice has been described (Lo Sicco et al., 2015). It has been shown how the injury signals were sufficient to specifically direct CH cell recruitment toward the fractured bone and how the peculiar local environment guided CH cell differentiation and appropriate integration into the specific tissue.

These different preclinical animal models have shown that small numbers of progenitor cells derived from the systemic circulation participate in the bone healing process (Hadjiargyrou and O’Keefe, 2014). Less amount of work has been performed to study the involvement of progenitor cells in humans, and the results obtained were controversial. Although some papers indicate that no circulating mesenchymal osteoprogenitors were detectable neither in healthy subjects nor in patients with end-stage renal or liver disease or in heart transplant patients (Hoogduijn et al., 2014), COPs were identified in the blood of a single patient with multiple fractures (Hoogduijn et al., 2014). The authors suggested that disruption of bone marrow, as a result of skeletal injury, could have allowed egress of mesenchymal progenitors into the circulation (Hadjiargyrou and O’Keefe, 2014). However, several cell types, other than classical mesenchymal progenitors, have been described to undergo osteogenic differentiation and migrate toward an injured bone under the action of appropriate stimuli (Szulc, 2016). A population of circulating cells with a myeloid origin expressing osteocalcin and bone alkaline phosphatase (OC+BAP+) has been demonstrated to possess an osteogenic activity *in vitro* and *in vivo*. Interestingly, the percentage of myeloid OC+BAP+ cells was higher in peripheral blood and bone marrow of type 2 diabetic patients, and in diabetic carotid endarterectomy specimens, a higher degree of calcification and amounts of OC and BAP-expressing cells were detected in the α-smooth muscle actin-negative areas surrounding calcified nodules, where CD68+ macrophages colocalized (Fadini et al., 2011). The increased percentage of COPs in pathological conditions was evidenced also in other clinical studies and it is supposed to be “linked to the presence of vascular damage, such as arterial stiffness and aortic calcification” (Pal et al., 2010; Pirro et al., 2011; Rattazzi et al., 2016). However, in 2005, Eghbali-Fatourechi et al. (2005) showed that the presence of a population of osteoblast-lineage cells circulating in physiological condition endowed with expression of markers of bone formation and markedly increasing during pubertal growth, thus representing a previously unknown component of bone formation process (Table 1).

## Conclusion

In recent years, significant advances have been accomplished in the comprehension of endogenous mechanisms promoting bone repair. Despite these advances, a huge confusion in endogenous COP identification still exists. This misperception could be due to various causes, such as the low understanding of the molecular mechanisms leading to the mobilization of progenitor cells from the niche in which they physiologically reside, the rate of their differentiation, and last but not least their prospective heterogeneity. In any case, altogether, the studies herein reviewed show the great potential of the endogenous repair mechanisms, envisaging new ways of thinking and new ways of moving forward in regenerative medicine.

## Author Contributions

CS and RT conceived the idea of this mini review and wrote the Abstract, Introduction, and Conclusion. RT revised and finalized the mini review.

## Conflict of Interest Statement

The authors declare that all financial, commercial or other relationships that might be perceived by the academic community as representing a potential conflict of interest are disclosed.
